# Association of childhood trauma with cognitive function in healthy adults: a pilot study

**DOI:** 10.1186/1471-2377-10-61

**Published:** 2010-07-14

**Authors:** Matthias Majer, Urs M Nater, Jin-Mann S Lin, Lucile Capuron, William C Reeves

**Affiliations:** 1 Dept. of Psychiatry & Behavioral Sciences, Emory University School of Medicine, Atlanta, GA, USA; 2 Chronic Viral Diseases Branch, Coordinating Center for Infectious Diseases, Centers for Disease Control& Prevention, Atlanta, GA, USA; 3 Laboratory of Psychoneuroimmunology, Nutrition and Genetics, INRA-University Victor Segalen Bordeaux 2, Bordeaux, France

## Abstract

**Background:**

Animal and human studies suggest that stress experienced early in life has detrimental consequences on brain development, including brain regions involved in cognitive function. Cognitive changes are cardinal features of depression and posttraumatic stress disorder. Early-life trauma is a major risk factor for these disorders. Only few studies have measured the long-term consequences of childhood trauma on cognitive function in healthy adults.

**Methods:**

In this pilot study, we investigated the relationship between childhood trauma exposure and cognitive function in 47 healthy adults, who were identified as part of a larger study from the general population in Wichita, KS. We used the Cambridge Neuropsychological Test Automated Battery (CANTAB) and the Wide-Range-Achievement-Test (WRAT-3) to examine cognitive function and individual achievement. Type and severity of childhood trauma was assessed by the Childhood Trauma Questionnaire (CTQ). Data were analyzed using multiple linear regression on CANTAB measures with primary predictors (CTQ scales) and potential confounders (age, sex, education, income).

**Results:**

Specific CTQ scales were significantly associated with measures of cognitive function. Emotional abuse was associated with impaired spatial working memory performance. Physical neglect correlated with impaired spatial working memory and pattern recognition memory. Sexual abuse and physical neglect were negatively associated with WRAT-3 scores. However, the association did not reach the significance level of p < 0.01.

**Conclusions:**

Our results suggest that physical neglect and emotional abuse might be associated with memory deficits in adulthood, which in turn might pose a risk factor for the development of psychopathology.

## Background

Traumatic experiences early in life, such as child abuse, neglect and parental loss, are major risk factors for the development of a range of psychiatric and somatic disorders in adulthood, including depression, posttraumatic stress disorder (PTSD), and chronic fatigue syndrome (CFS) [1-3]. There is ample evidence from animal models and human studies that adverse experience early in life, during periods of heightened brain plasticity, permanently programs the development of multiple brain circuits involved in the processing of environmental stimuli and the regulation of behavioral, autonomic, and endocrine responses to stress [4]. Studies in humans have shown that several cardinal biological features of depression or CFS are secondary to early-life trauma and might reflect vulnerability for the development of these disorders [5,6].

Several of the disorders that have been linked to childhood trauma are characterized by profound cognitive impairment, including depression, PTSD, and CFS. There is direct evidence from animal models that early-life stress induces structural, functional, and epigenetic changes in brain regions involved in cognition, most notably the hippocampus [see [4]]. Small hippocampal volume is associated with cognitive impairment, specifically memory deficits, in patients with depression or PTSD [7-9]. While hippocampal impairment may be the result of toxic effects of chronic cortisol overexposure or increased glucocorticoid sensitivity [4], more recent evidence suggests that small hippocampal volume might be a preexisting risk factor [4,4,9,10]. Of note, hippocampal volume loss in depression has been associated with early-life trauma [11]. It might be conceivable that cognitive impairment in these disorders is linked to early-life trauma. Whether or not early-life stress is associated with long-term cognitive deficits in humans is largely unknown.

Maternal separation of rats results in enduring hippocampal dysfunction, including impaired memory and spatial learning [12-16]. Juvenile rats exposed to a platform stress perform poorer on a spatial learning task in adulthood than control rats [17]. These cognitive deficits persist through late adulthood and early aging [18]. Similar results have been obtained in studies with non-human primates. Rhesus macaque monkeys reared in isolation exhibit significant deficits in learning and memory [19-21]. Few studies have investigated associations between childhood adversity and cognitive function in humans. Infants and toddlers who have been abused, neglected or exposed to multiple medical and surgical procedures often exhibit deficits in cognitive, language, and motor skills [22,23]. Palmer et al. [24] found profound impairments in cognition, including intellectual development delays and language and psychomotor deficiencies in sexually abused children. Abused or neglected children also have a higher risk for poor academic achievement [25]. On the other hand, two studies comparing groups of trauma-exposed and non-exposed children and adolescents on cognitive outcome measures found that trauma exposure was not associated with lower estimates of intelligence [26] or memory and learning deficits [27]. Given the limited information and inconsistency of the human literature, more human research is needed that examines cognitive performance of persons exposed to traumatic events early in life.

The aim of this study was to assess the association between childhood trauma exposure and cognitive function in healthy adults. We hypothesized that exposure to childhood trauma would be significantly associated with impairment in cognition, specifically hippocampus-related memory function, and that childhood trauma would be significantly associated with academic underachievement. To test these hypotheses we measured childhood trauma exposure, neurocognitive function, and level of academic achievement in a group of healthy adults (without concurrent psychiatric illness) randomly selected from the Wichita, KS population. These subjects were recruited as controls for a larger CDC study [28]. We intended this pilot study to provide impetus for the development of future case-control matched studies that further scrutinize the association between childhood stress and adult cognitive function.

## Method

### Participants

This study adhered to U.S. Department of Health and Human Services human experimentation guidelines and received Institutional Review Board approval from the CDC and collaborating institutions. All participants gave informed consent.

Participants were a subset of 47 healthy adults, selected from a sample of 227 individuals participating in a clinical study of chronic fatigue syndrome (CFS) [for details see, [28]]. The 47 healthy subjects had been randomly selected from the population in a random-digit dialing telephone survey, followed by clinical examination. The subjects included here are healthy controls with no concurrent medical or psychiatric illness. These subjects were identified from the population to match CFS cases in a larger study (not reported here) based on age, sex, race, and body mass index. Subjects were free of current and lifetime psychiatric diagnoses (mood disorders, psychosis, substance use disorders, anxiety disorders, somatoform disorders and eating disorders), as verified by licensed and specifically trained psychiatric interviewers who administered the Diagnostic Interview Schedule (DIS) for Axis I psychiatric disorders [29]. To exclude medical conditions, participants underwent a standardized physical examination conducted by a specifically trained physician. The physician also reviewed past medical history, review of systems and medication; then, following the standardized physical examination, s/he evaluated specific systems in more detail, as warranted. Finally, participants provided blood and urine for routine analysis; the study physician reviewed results the following morning and considered them in terms of the previous evening's physical examination. All subjects were free of medical conditions and psychotropic medications. Forty-seven healthy adults were included in the current study. All subjects gave informed consent.

### Assessment of Childhood Trauma

We assessed exposure to childhood trauma before onset of puberty by the Short-Form of the Childhood Trauma Questionnaire (CTQ) [30]. This self-report questionnaire measures five dimensions of childhood trauma experience, including emotional, physical, and sexual abuse, and emotional and physical neglect. Examples of questions are: "People in my family called me stupid, lazy or ugly" (Emotional Abuse-item); "People in my family hit me so hard that it left bruises or marks" (Physical Abuse-item); "Someone threatened to hurt me or tell lies about me unless I did something sexual with them" (Sexual Abuse-item); "I knew there was someone to take care of me and protect me" (Emotional Neglect-inverse item); "There was someone to take me to the doctor if I needed it" (Physical Neglect-inverse item). Each subscale consists of five items rated on a 5-point Likert scale ranging from "never true" to "very often true". Such Likert-type items create dimensional scales providing quantitative scores that have enhanced reliability and maximized statistical power [30]. In addition to dimensional scores, cutoff scores for none-low, low-moderate, moderate-severe, and severe-extreme exposure are provided for each scale. Moderate-severe cutoff scores for each subscale are >= 13 for Emotional Abuse; >= 10 for Physical Abuse; >= 8 for Sexual Abuse; >= 15 for Emotional Neglect; and >= 10 for Physical Neglect. Being identified as positive for a category corresponds with endorsing a substantive number of experiences as "often true". The Childhood Trauma Questionnaire has good internal consistency (0.63-0.95) and criterion-related validity (0.50-0.75) in clinical and community samples. Based on a sample of 223 individuals enrolled in the initial study [28], the internal consistency coefficients (Cronbach's alpha) for the five subscales range from 0.68 to 0.92. Convergent reliability with therapist assessments of abuse histories is high. Based on the current sample of the 47 healthy subjects, the internal consistency coefficients (Cronbach's alpha) for the five subscales range from 0.41 to 0.89.

### Assessment of Depression and Anxiety

Although subjects were by definition healthy and free of any psychiatric disorder, verified by DIS, we did obtain depression and anxiety ratings to ascertain that sub-threshold symptoms would not influence results. We administered standard self-report dimensional rating scales including the Zung Self rating Depression Scale [31] and the Spielberger State-Trait-Anxiety Inventory [32],

### Neuropsychological Assessment

We utilized the reading subtest of the Wide-Range-Achievement-Test (WRAT-3) [33] to assess academic achievement. We used the Cambridge Neuropsychological Test Automated Battery (CANTAB) to evaluate cognitive function [34]. Execution of the CANTAB required about 60 minutes, involved 7 tests designed to assess a broad range of cognitive functions. The order in which tests were presented was counterbalanced between subjects. Tests included:

#### Memory

a) The *Spatial Working Memory *task measures subjects' ability to retain spatial information and to manipulate remembered items in working memory. The Spatial Working Memory task requires that subjects find blue tokens in a series of displayed boxes. Three different types of errors are possible: (1) 'Between' errors, which occurred when subjects, on any particularly search, revisits a box in which a token has previously been found; (2) 'within' errors, which are committed when the subject returned to an empty box before finding the next token in that search sequence; and (3) 'double' errors, which were a combination of between and within errors. These occurred when a subject repeated a between error before finding the next token, again in that particular search sequence. The number of double errors in the 4-, 6-, and 8-boxes as well as the total number of double errors were used as performance indices. A strategy score derived from the number of search sequences in the 4-, 6-, and 8-boxes was also used as performance index. The strategy score retraced the 'route' previously used by subjects in searching through the spatial array of boxes. The lower the strategy score, the more efficient was the subject.

b) The *Pattern Recognition Memory *task employs a delayed match-to-sample paradigm to assess recognition memory for visual patterns. The participant is presented with a series of 12 geometric patterns of varying colors one after another (encoding phase). During the test phase, two stimuli are presented side by side, and the participant is instructed to choose the one that was seen during the previous encoding phase. The Pattern Recognition Memory task is sensitive to temporal or hippocampal dysfunctions [35]. Percent correct responses (successful recognition) and response latency for correct responses were used as performance indices.

c) During the *Spatial Recognition Memory *task, the computer presents a series of five white squares in random locations on the computer screen (encoding phase). During the test phase the computer presents five pairs of squares, one of which is in the same location on the screen as one of the previously presented squares from the encoding phase. The participant must select the square that is in the same location. Spatial Recognition Memory has its putative neural circuitry located in the frontal lobe [35]. As for Pattern Recognition Memory, percent correct responses (successful recognition) and response latency for correct responses were used as performance indices for the Spatial Recognition Memory task.

#### Executive Function

a) The *Stockings of Cambridge *task assesses subject's ability to engage in spatial planning/problem solving. It makes substantial demands on executive function and is sensitive to frontal-lobe deficits [36]. The subject is shown two displays containing colored balls, and has to use the balls in the lower display to copy the pattern shown in the upper one. The time taken to copy the pattern (including the time taken to make the first move after the presentation of the display (initial thinking time) and the time spent thinking about a problem during its execution (subsequent thinking time)), the number of perfect solutions (problems solved in a minimum of moves), and the average number of moves required to solve the problem were taken as the subject's planning abilities.

b) The *Intra/Extra Dimensional Shift *task is a test of rule acquisition and reversal, featuring visual discrimination and shifting of attention. Two stimuli (one correct, one incorrect) in the form of shapes and lines are presented in four possible locations on the computer screen. First, the subject has to maintain attention to different examples of stimuli within the same dimension (shapes), while distracting stimuli of different dimensions are present (lines) (intra-dimensional shifts). Second, the subject has to shift attention to the previous irrelevant dimension (lines) and ignore the previous relevant dimension (shapes) (extra-dimensional shifts). Subjects progress through the test by satisfying a set of criteria of learning at each stage (nine stages in total). The total number of stages achieved and the errors made at the extra-dimensional shift served as performance indices.

#### Psychomotor Speed and Sustained Attention

a) The *Reaction Time *task utilizes simple and five-choice reaction time tasks to measure psychomotor speed. This test is divided into 5 stages requiring increasingly complex chains of responses and providing distinction between reaction (or decision) time and movement latencies. Five-choice reaction and movement times were taken as performance indices.

b) The *Rapid Visual Information Processing *task is a 4-min visual sustained attention task with a small working memory component. Subjects press a response pad when they detect any one of three number sequences in a continuous presentation of numbers. Performance accuracy was estimated using the metric A' (a signal detection measure of sensitivity to the target regardless of response tendency, ranging from 0.00 to 1.00; bad to good); performance speed was assessed by the mean latency for correct responses.

### Statistical Analysis

Data were analyzed by multiple linear regression models on WRAT-3 and CANTAB measures with primary predictors (CTQ scales) and potential confounders (age, sex, education, income). For consideration of Type I error rates and the sample size of the study, we chose a significance level of *p *< 0.01 for associations between the primary predictors (CTQ scales) and the primary outcome variables (CANTAB measures and WRAT-3). Data were analyzed using SPSS 15.0.

## Results

Sample characteristics are shown in Table 1. As can be seen in the Table, mean CTQ scores across all subjects were in the none-mild range. Nevertheless, a proportion of 25.5% of subjects had experienced at least one type of maltreatment that scored above the cut-off for moderate-severe abuse. The most common form of reported maltreatment was emotional abuse. Mean depression ratings were in the normal range (i.e. no depression) and state and trait anxiety ratings were low (see Table 1). None of the subjects had a depression score in the clinically significant range; none of the subjects had state or trait anxiety above the population average.

**Table 1:** Sample Characteristics and Early Life Stress (n = 47)

Variable	
Age (SE; range)	51.51 (1.22; 31-69)
Sex	7 male, 40 female
Race	44 white, 2 black, 1 other
Education	
≤ High school or vocational tech diploma	20
Associate degree, RN diploma or college	
Income ($/year)^a^	27
20,000 - 40,000	
> 40,000	13
WRAT-3 standard score (SE)	33
CTQ Total score (5-125) (Mean (SE))	33.7 (1.34)
Emotional abuse (5-25) (Mean (SE))	7.7 (.516)
Physical abuse (5-25) (Mean (SE))	6.36 (.259)
Sexual abuse (5-25) (Mean (SE))	5.85 (.373)
Emotional neglect (5-25) (Mean (SE))	7.9 (.479)
Physical neglect (5-25) (Mean (SE))	5.85 (.228)
CTQ Exposures ^b^, n (%)	12 (25.5%)
Emotional abuse	8 (17.02%)
Physical abuse	4 (8.51%)
Sexual abuse	3 (6.38%)
Emotional neglect	2 (4.26%)
Physical neglect	2 (4.35%)
Self-Rating Depression Scale (Mean (SE))	37.5 (.106)
Spielberger State Anxiety (Mean (SE))	26.1 (.832)
Spielberger Trait Anxiety (Mean (SE))	27.5 (.772)

*Note*. *SE *= standard error of the mean; WRAT-3 = Wide Range Achievement

Test 3; CTQ = Childhood Trauma Questionnaire; ^a ^Information on income is missing for one participant; ^b ^Using the cut-off values from the study of Bernstein & Fink, 1998 (see Methods section).

### Achievement

After adjustment for age, sex, education, and income, multiple linear regression analysis revealed an association between the sexual abuse and the physical neglect scores of the CTQ and the WRAT-3 standard score (sexual abuse: adjusted *B *= -6.18, SE = 2.86, *p *= 0.03; physical neglect: adjusted *B *= -9.28, SE = 3.97, *p *= 0.02). However, the association did not achieve a more conservative statistical significance level of 0.01 for multiple separate hypothesis testing. The association indicated that more exposure to sexual abuse or physical neglect in childhood was associated with worse performance in the WRAT-3 test as an adult.

### Memory

Multiple linear regression analyses revealed three significant associations between CTQ scale scores and CANTAB measures of memory (Table 2). The emotional abuse score was significantly associated with the number of double errors in the Spatial Working Memory test. Furthermore, there was a significant association between the physical neglect score and the number of double errors in the Spatial Working Memory test and the latency for a correct response in the Pattern Recognition Memory test. The more exposure to these two different types of childhood trauma, the worse was the memory performance.

**Table 2:** Associations of exposure to childhood trauma with CANTAB measures of memory

		**CTQ **^**a **^**scales**				
	
**CANTAB **^b ^**variable**	Mean (SE)	Emotional abuse, ** adjusted *B *(SE)**^**c**^	Physical abuse, ** adjusted *B *(SE)**^**c**^	Sexual abuse, ** adjusted *B *(SE)**^**c**^	Emotional neglect, ** adjusted *B *(SE)**^**c**^	Physical neglect, ** adjusted *B *(SE)**^**c**^
**Spatial Working Memory**						
Strategy score	34.18 (0.53)	-0.07 (0.86)	-0.23 (1.80)	0.74 (1.12)	-0.25 (0.93)	0.06 (1.84)
Standardized B^**c**^		-0.01	-0.03	0.11	-0.05	0.00
# Double errors (total)	2.74 (0.74)	3.44 (1.05)**	5.92 (2.30)*	0.56 (1.54)	0.80 (1.27)	6.59 (2.31)**
Standardized B^**c**^		0.48	0.41	0.07	0.10	0.40

**Spatial Recognition Memory**						
% Correct	84.15 (1.08)	-0.31 (1.72)	-4.38 (3.55)	0.91 (2.25)	-0.25 (1.87)	0.01 (3.69)
Standardized B^**c**^		-0.03	-0.21	0.06	-0.02	0.00
Mean correct latency (ms)	2567.11 (110.29)	-39.01 (170.51)	-225.73 (357.48)	-129.08 (222.88)	32.12 (185.40)	393.77 (361.68)
Standardized B^**c**^		0.31	-0.11	-0.09	0.03	0.16

**Pattern Recognition Memory**						
% Correct	90.34 (1.48)	-5.49 (2.20)*	-9.90 (4.73)	4.33 (3.03)	-1.29 (2.56)	-2.54 (5.08)
Standardized B^**c**^		-0.39	-0.35	0.22	-0.08	-0.08
Mean correct latency (ms)	2304.76 (123.25)	460.12 (180.88)	717.64 (394.50)	-187.78 (254.00)	238.08 (208.55)	1213.18 (372.90)**
Standardized B^**c**^		0.39	0.30	-0.11	0.19	0.45

^a ^Childhood Trauma Questionnaire; ^b ^Cambridge Neuropsychological Test Automated Battery; ^c ^Regression coefficient B and standardized coefficient B, adjusted for age, sex, education, income (SE: standard error of the mean); ** *p *< 0.01, *p = 0.01

### Executive Functions

As shown in Table 3, there were no significant associations found between CTQ scale scores and CANTAB measures of executive functions. The two measures of the Intra/Extra Dimensional Shift task, assessing rule acquisition and reversal, featuring visual discrimination and shifting of attention, were not statistically significantly associated with any of the CTQ scale scores. Similarly, there were no significant associations between the different CTQ scales and reasoning and planning abilities, as measured by the Stockings of Cambridge task.

**Table 3:** Associations of exposure to childhood trauma with CANTAB measures of executive functions

		**CTQ **^**a **^**scales**				
	
**CANTAB **^b ^**variable**	Mean (SE)	Emotional abuse, ** adjusted *B *(SE)**^**c**^	Physical abuse, ** adjusted *B *(SE)**^**c**^	Sexual abuse, ** adjusted *B *(SE)**^**c**^	Emotional neglect, ** adjusted *B *(SE)**^**c**^	Physical neglect, ** adjusted *B *(SE)**^**c**^
**Intra/Extra Dimensional Shift**						
# Stages completed	8.43 (0.14)	0.05 (0.23)	-0.53 (0.48)	-0.73 (0.28)	-0.35 (0.25)	-0.71 (0.49)
Standardized B^**c**^		0.04	-0.19	-0.38	-0.24	-0.23
# EDS errors	9.66 (1.44)	-0.68 (2.36)	1.90 (4.96)	0.01 (3.10)	-1.32 (2.56)	0.81 (5.08)
Standardized B^**c**^		-0.05	0.07	0.00	-0.09	0.03

**Stockings of Cambridge**	8.83 (0.31)	-0.42 (0.45)	-1.16 (0.95)	0.93 (0.58)	-0.16 (0.50)	0.52 (0.98)
Total # perfect solutions		-0.14	-0.19	0.23	-0.05	0.08
Standardized B^**c**^	5.37 (0.14)	0.23 (0.19)	0.30 (0.41)	-0.11 (0.26)	0.23 (0.21)	0.08 (0.42)
4-Move Problems		0.17	0.11	-0.06	0.16	0.03
Average # moves	11624.73	-2311.04	-3940.05	-748.42	-2716.01	1787.55
Standardized B^**c**^	(1055.29)	(1423.33)	(3030.73)	(1923.94)	(1538.53)	(3148.11)
ITT (ms)		-0.24	-0.20	-0.06	-0.26	0.08
Standardized B^**c**^	2071.30 (321.45)	106.29 (460.81)	-551.89 (966.98)	36.82 (604.83)	-6.59 (501.23)	212.98 (991.26)
STT (ms)		0.03	-0.09	0.01	-0.00	0.03
Standardized B^**c**^						
5-Move Problems	6.40 (0.20)	0.28 (0.31)	0.92 (0.65)	-0.67 (0.40)	-0.01 (0.34)	-0.17 (0.68)
Average # moves		0.14	0.23	-0.25	-0.00	-0.04
Standardized B^**c**^	13879.70	-1568.09	-8073.42	-230.52	621.74	-115.66

ITT (ms)	(1389.96)	(2185.76)	(4452.37)	(2885.29)	(2389.13)	(4731.53)
Standardized B^**c**^		-0.12	-0.30	-0.01	0.04	-0.00
STT (ms)	1021.84 (161.05)	210.91 (253.29)	626.28 (528.72)	-498.01 (325.73)	-199.99 (275.89)	-212.60 (548.48)
Standardized B^**c**^		0.14	0.20	-0.23	-0.12	-0.06

^a ^Childhood Trauma Questionnaire; ^b ^Cambridge Neuropsychological Test Automated Battery; ^c ^B and standardized coefficient B adjusted for age, sex, education, income (SE: standard error of the mean); ITT: initial thinking time; STT: subsequent thinking time.

### Psychomotor Speed and Sustained Attention

Performance on the Reaction Time task, as measured by five-choice reaction and five-choice movement times, was not significantly associated with the CTQ scales, nor were there significant associations between the CTQ scale scores and performance as measured by signal A' and mean latencies to correct responses in the Rapid Visual Information Processing test (Table 4).

**Table 4:** Associations of exposure to childhood trauma with CANTAB measures of psychomotor speed and sustained attention

		**CTQ **^**a **^**scales**				
	
**CANTAB **^b ^**variable**	Mean (SE)	Emotional abuse, ** adjusted *B *(SE)**^**c**^	Physical abuse, ** adjusted *B *(SE)**^**c**^	Sexual abuse, ** adjusted *B *(SE)**^**c**^	Emotional neglect, ** adjusted *B *(SE)**^**c**^	Physical neglect, ** adjusted *B *(SE)**^**c**^
**Reaction Time**						
5-choice reaction time (ms)	396.20 (8.66)	-3.86 (14.07)	-16.38 (29.53)	-7.07 (18.44)	-15.34 (15.11)	14.16 (30.20)
Standardized B^**c**^		-0.05	-0.10	-0.06	-0.17	0.07

5-choice movement time (ms)	479.97 (16.28)	60.21 (24.31)	70.17 (53.84)	-34.80 (33.80)	19.63 (28.21)	95.84 (54.07)
Standardized B^**c**^		0.39	0.23	-0.16	0.12	0.27

**Rapid Visual Information Processing**						
Mean latency (ms)	548.43 (19.58)	7.06 (27.89)	33.45 (58.87)	-31.36 (36.28)	15.57 (31.28)	40.50 (59.82)
Standardized B^**c**^		0.04	0.09	-0.12	0.08	0.10
A'		-0.016 (0.010)	-0.023 (0.022)	-0.016 (0.013)	-0.026 (0.011)	-0.021 (0.022)
Standardized B^**c**^	0.90 (0.01)	-0.25	-0.17	-0.18	-0.37	-0.14

^a ^Childhood Trauma Questionnaire; ^b ^Cambridge Neuropsychological Test Automated Battery; ^c ^B and standardized coefficient B adjusted for age, sex, education, income (SE: standard error of the mean).

## Discussion

This pilot study found significant associations between level of childhood trauma exposure and cognitive performance in CANTAB measures of long-term and working memory in a group of healthy adults, with no significant symptoms of depression or anxiety, that were randomly selected from the general population. Healthy adults with high exposure to emotional abuse, the most common form of reported maltreatment in this sample, exhibited a higher error rate in the Spatial Working Memory test. Furthermore, individuals with high levels of exposure to physical neglect showed a higher error rate in the Spatial Working Memory test and prolonged latency to a correct response in the Pattern Recognition Memory test. Finally, we found a (less significant) association between level of exposure to sexual abuse or physical neglect and lower scores in the reading subtest of the WRAT-3, indicating less academic achievement in traumatized subjects. Our results add to a growing literature that supports a relationship between childhood trauma exposure and the development of cognitive dysfunction in children and poor academic achievement [22-25]. The current findings suggest that memory deficits are specifically associated with childhood trauma exposure in healthy adults.

Negative associations between childhood trauma exposure and cognitive performance were found in the domains of long-term and working memory. Working memory refers to the structures and processes used for temporarily storing and manipulating information. Long-term memory differs structurally and functionally from working memory. It holds information from a few minutes to decades [37]. Participants with higher levels of physical neglect showed longer response latencies in the Pattern Recognition Memory task, a test for long-term memory. This deficit cannot be explained by reduced elemental speed of cognitive processing, since there was no significant association between physical neglect and the response time measures in the Reaction Time task. Therefore, our results suggest a specific deficit in the ability to judge the prior occurrence of visual patterns (long-term memory) in subjects with higher levels of exposure to physical neglect. In Spatial Working Memory, we found that subjects with more exposure to physical neglect or emotional abuse had a higher rate in double errors. Efficient solving of problems in the spatial working memory test requires remaining highly attentive, using memory skills to remember previously selected and targeted locations, and developing and maintaining strategies to organize each search. Attentional problems did not appear to affect performance in Spatial Working Memory since there was no association between level of physical neglect or emotional abuse and performance in the Rapid Visual Information Processing task, a test of sustained attention. Organizational abilities were also intact in subjects with higher levels of childhood trauma since we found no relationship between the strategy scores in the Spatial Working Memory task or the scores in the Stockings of Cambridge task and physical neglect or emotional abuse. Therefore the higher error rate in the Spatial Working Memory task in subjects with higher levels of physical neglect or emotional abuse presumably reflects a pure memory deficit in these subjects. Although no brain activity was measured in this study, one might speculate that the observed memory deficits are linked to altered structure of function of brain regions. Animal models of early-life stress suggest that medial temporal structures are affected. Rats, exposed to a period of early-life stress, show late-onset, selective deterioration of cognitive performance in a hippocampus-involving object recognition memory task [8]. Clinical evidence is brought by studies of patients with hippocampal damage. Patients with temporal lobe or amygdala-hippocampectomy damage show cognitive deficits in pattern recognition memory compared to those with frontal lobe excision [35]. Furthermore, patients suffering from senile dementia of the Alzheimer type show impairments in spatial working memory that are accompanied by evidence of an intact strategy approach to the task, suggesting a pure memory deficit associated with hippocampal impairment in these patients [38]. Of note, memory deficits are core features of depression and PTSD, and these disorders are also associated with hippocampal damage [4,7-9]. Hippocampal volume loss in depression has been associated with early life trauma [11]. Taken together, our study results suggest that exposure to emotional neglect or physical abuse is associated with cognitive underperformance in tasks that involve the hippocampus.

Cognitive deficits after childhood trauma could be a direct consequence of the effects of trauma on the brain or could occur as a result of psychiatric illness, alcohol and substance abuse, or medical illness, which are associated with childhood trauma. Especially, the deficits in working and long-term memory found in this study are quite characteristic for patients with depression [4,7]. In our study, however, cognitive deficits were found in adult survivors of childhood trauma who did *not *suffer a current medical or psychiatric illness (including major depressive disorder) and did *not *have a history of alcohol or substance abuse. Subjects were also free of subsyndromal depression and anxiety as tested using rating scales. Hence, the cognitive deficits linked to childhood trauma are not secondary to depression or other psychiatric or medical illnesses.

There are several limitations of the present study. First, sample size is very small, which might have led to false positive or false negative results due to outliers. Replication of our findings in larger samples is necessary. Such studies might include subjects recruited based on childhood trauma in order to obtain more cases with severe trauma and larger cells for different trauma types, which would allow for comparisons of cognitive function between groups. Second, we relied on retrospective and uncorroborated self-reports of childhood experiences. Problems concerning the credibility of self-reported childhood trauma include simple forgetting, non-awareness, nondisclosure, and reporting biases due to mood states. However, the use of validated psychometric instruments increases validity of self-reported data [39]. Third, we did not consider effects of adulthood trauma and life stress that might mediate or moderate the relationship between childhood adversity and cognitive dysfunction, since individuals with early adverse experience more frequently experience adulthood stresses, and moreover, are sensitized to the effects of such stressors [40]. Fourth, owing to the cross-sectional design, we cannot determine whether cognitive dysfunction might have preceded early adversity. Finally, it must be noted that we studied a sample comprising subjects who had significant exposure to early-life trauma but remained healthy. Indeed, there is a substantial amount of resilience after early-life stress [41,42]. One must consider the possibility that our findings reflect cognitive changes specific to resilient persons with early life trauma, which might be different from those persons with early-life trauma who go on to develop a disorder.

## Conclusions

Results of this pilot study should be considered as preliminary. Our observations lend support for the hypothesis that exposure to childhood trauma, especially emotional abuse and physical neglect, leads to problems in long-term and working memory in adulthood. Longitudinal studies are needed to provide more information on the causal relationship between childhood trauma and memory deficits, and to systematically evaluate developmental trajectories as well as mediators and moderators of this relationship. These studies might consider maternal education and mother-child interactions as moderator variables as developmental psychologists have reported that these variables are partially predictive of the cognitive performance of children [43]. Future studies might also consider the potential role of socioeconomic status in contributing to the cognitive deficits. Our results might have important clinical implications as cognitive impairment is a key factor in many psychiatric disorders, such as major depression or post-traumatic stress disorder. Cognitive deficits should be addressed in the treatment of victims of childhood trauma, independent of their being concurrently diagnosed with a psychiatric condition. Neuropsychological training should be implemented in a comprehensive intervention strategy for individuals with childhood trauma. Furthermore, early intervention may prevent the long-term deficits in memory function, and hence academic performance in maltreated children.

## Competing interests

The authors declare that they have no competing interests.

## Authors' contributions

All authors read and approved the final manuscript. WCR was principal investigator of the study. MM, UMN, JSL, LC, and WCR wrote the manuscript. All authors contributed to the manuscript. WCR designed the study protocol and supervised data collection during the study. WCR supervised the conduct of clinical studies and data collection. MM and JSL conducted the statistical analyses.

## Pre-publication history

The pre-publication history for this paper can be accessed here:

http://www.biomedcentral.com/1471-2377/10/61/prepub
